# Supplementary material for: Sulfasalazine as an Immunomodulator of the Inflammatory Process during HIV-1 Infection
Source: Int J Mol Sci. 2019 Sep 11;20(18):4476. doi: 10.3390/ijms20184476 (PMC6770882; doi:10.3390/ijms20184476)
Supplement: Supplementary file 1 [file ijms-20-04476-s001.pdf]

Supplementary Data

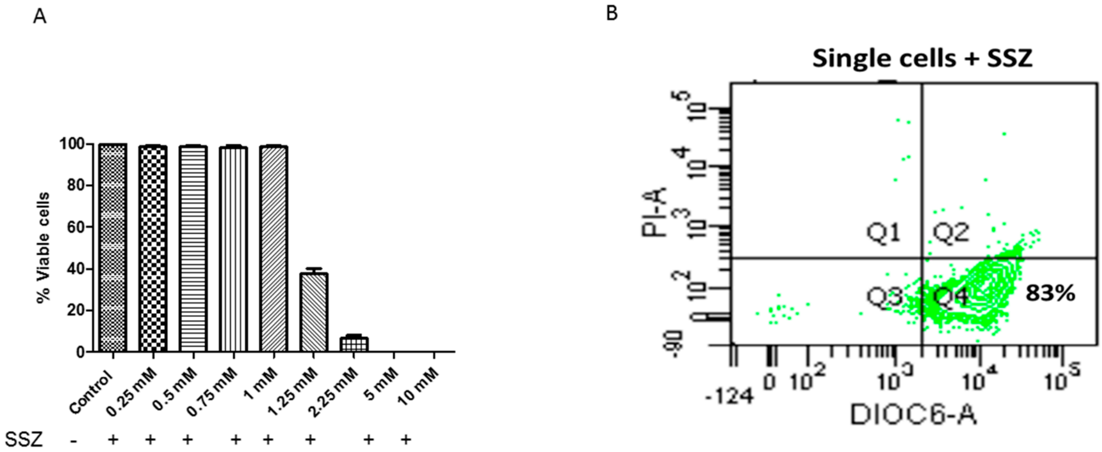

Figure S1. Determination of cell viability in PBMC treated with SSZ. The cell viability was estimated by trypan blue exclusion assay (**A**) after treatment with SSZ (0.25 to 10 mM). The cell viability in PBMC treated with 1 mM SSZ was confirmed by DIOC6 / PI staining and flow cytometry. The percentage of viable cells was determined by gating on PI-negative and DiOC6-bright cells (lower right quadrant).

Table S1. Sequences used for mRNA amplification of inflammasome-related gene.

Gen	Sequence of Primers 5'–3'	Annealing Temperature	Product Length (bp)
**IL-1β**	Fw: CTTTGCCGATCCGCCGC<br>Rv: ATCACGCCCTGGTGCCTGG	60 °C	174
**IL-18**	Fw: ATGGCTGCTGAACCAGTAGAAG<br>Rv: CAGCCATACCTCTAGGCTGGC	62 °C	292
**NLRP3**	Fw: AGCACCAGCCAGAGTCTAAC<br>Rv: CCCCAACCACAATCTCCGAAT	57 °C	123
**NLRP1**	Fw: CTATACTTCCCGAGGCATCCTT<br>Rv: GGTCTTGGAAGTCAGTGTGAGT	56 °C	301
**NLRC4**	Fw: CTCTCATGGTGGAAGCCAGTCC<br>Rv: ACAGAGACTTGACTATGTAATCC	56 °C	301
**AIM2**	Fw: AAGCGCTGTTTGCCAGTTAT<br>Rv: CACACGTGAGGCGCTATTTA	55 °C	231
**ASC**	Fw: AACCCAAGCAAGATGCGGAAG<br>Rv: TTAGGGCCTGGAGGAGCAAG	62 °C	82
**Caspase-1**	Fw: CAAGGGTGCTGAACAAGG<br>Rv: GGGCATAGCTGGGTTGTC	60 °C	278
**Ubiquitin**	Fw: CCTTCAAACCACCTAAGGTTGC<br>Rv: GTGCTGGAGACCACTGTGATGG	58.7 °C	109
